# Palbociclib-Induced Pneumonitis: A Case Report and Review of the Literature

**DOI:** 10.7759/cureus.8929

**Published:** 2020-06-30

**Authors:** Saro Sarkisian, Christopher Markosian, Zeeshan Ali, Muhammad Rizvi

**Affiliations:** 1 Hematology and Oncology, Lehigh Valley Cancer Institute, Allentown, USA; 2 Hematology and Oncology, Rutgers New Jersey Medical School, Newark, USA

**Keywords:** hr+/erbb2− breast cancer, palbociclib, pneumonitis

## Abstract

The treatment of metastatic breast cancer has undergone significant changes in recent years. New classes of medications have been approved by the Food and Drug Administration (FDA) for use in clinical practice to extend progression-free survival and overall survival along with increasing response rate. Here, we present a case report of pneumonitis as a rare side effect of palbociclib in the treatment of metastatic hormone receptor-positive (HR+)/human epidermal growth factor receptor 2-negative (ERBB2−) breast cancer in addition to endocrine therapy. We also review the literature for other reports of pneumonitis during treatment with palbociclib. Through this case report and review of the literature, we aim to shed light on this rare side effect of palbociclib along with its successful management.

## Introduction

Breast cancer is the most commonly diagnosed cancer in women globally [1]. The vast majority of diagnosed breast cancers are hormone receptor-positive (HR+)/human epidermal growth factor receptor 2-negative (ERBB2−) [2]. The treatment of metastatic HR+/ERBB2− breast cancer is endocrine therapy until the cancer becomes endocrine resistant [3]. An oral aromatase inhibitor (AI) is used in the first-line setting for postmenopausal women, while subsequent lines of therapy involve other forms of endocrine therapy in addition to AIs.

Palbociclib is an oral cyclin-dependent kinase 4 and 6 (CDK4/6) inhibitor that can be incorporated in addition to endocrine therapy for the treatment of metastatic HR+/ERBB2− breast cancer [4-6]. Palbociclib can be used in the first line of treatment in combination with letrozole, an oral AI, or in a subsequent line in combination with fulvestrant, a selective estrogen receptor degrader [3,4]. Various grade 3 or 4 side effects were noted in the palbociclib-fulvestrant group of the PALOMA3 clinical trial and the palbociclib-letrozole group of the PALOMA-2 clinical trial [5,6]. A rare side effect of palbociclib that was not initially reported in these phase 3 clinical trials but has been described in the literature is pneumonitis [7-13].

We describe a rare case of palbociclib-induced pneumonitis in a patient with metastatic HR+/ERBB2− breast cancer. We also review the literature for cases of pneumonitis associated with palbociclib therapy.

## Case presentation

A 72-year-old female without significant past medical history presented to the hospital with progressive lumbar back pain that initially started a few months prior but worsened three days prior to her presentation. Her pain was radiating to her right leg and was associated with difficulty with ambulation. No urinary or stool incontinence was present. Of note, she felt a hard lump in her right breast a few months prior for which she did not seek medical attention. Prior to hospital presentation, she was treated symptomatically by her primary care physician with non-steroidal agents and a brief course of prednisone with only mild improvement in her symptoms. Her review of systems was negative except for the described complaints. Physical examination was notable for point tenderness in her lumbar area with a firm, immobile mass measuring about 6 cm in her right breast and right axillary lymphadenopathy.

**Figure 1:**
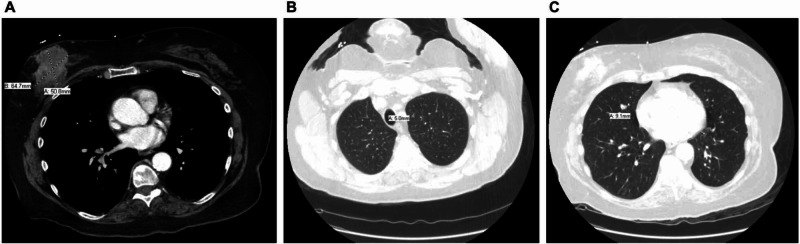
Various CT scan views display (A) right breast mass (axial view) and (B and C) pulmonary nodules (axial views).

**Figure 2:**
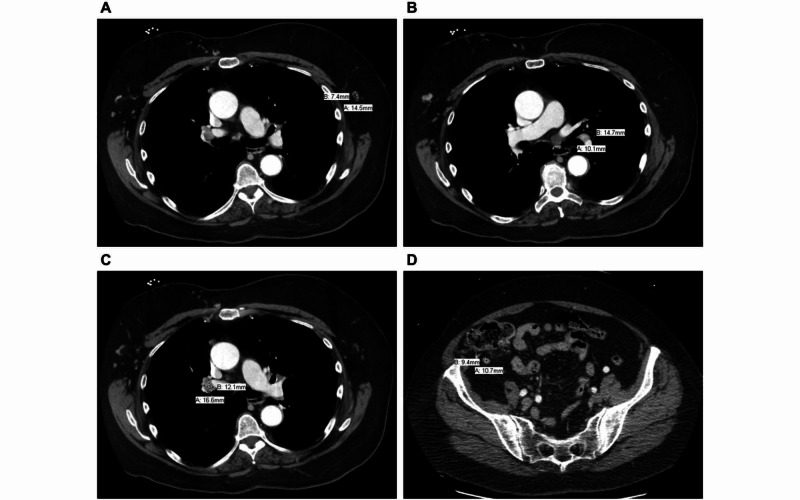
Various CT scan views display (A) axillary lymphadenopathy (axial view), (B) hilar lymphadenopathy (axial view), (C) mediastinal lymphadenopathy (axial view), and (D) mesenteric lymphadenopathy (axial view).

Her laboratory values were unremarkable. X-ray imaging of her lumbar back revealed a sclerotic density in her L2 vertebra. This was followed by MRI of her lumbar area, which revealed multiple bony metastases at every level between T11 and L5 along with a pathological compression fracture at L2 with moderate encroachment of the central canal at L2 to L3 with epidural extension of disease. A CT scan of her chest, abdomen, and pelvis with IV contrast revealed a right breast mass measuring 6.5 cm by 5.1 cm (Figure 1A), which was associated with pulmonary nodules (Figures 1B, 1C), along with axillary (Figure 2A), hilar (Figure 2B), mediastinal (Figure 2C), and mesenteric lymphadenopathy (Figure 2D), suggestive of metastatic disease with her known osseous metastases seen on MRI. During hospitalization, a core biopsy of the right breast mass was performed, which revealed HR+/ERBB2− disease. After stabilization from this acute event with palliative radiation therapy and pain medications, she was discharged with instructions for outpatient follow-up.

**Figure 3:**
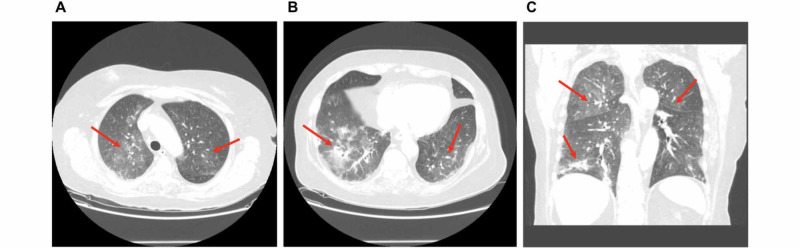
Various CT scan views display (A) ground-glass opacities in the bilateral upper lobes (axial view), (B) ground-glass opacities in the bilateral lower lobes (axial view), and (C) ground-glass opacities in the bilateral upper lobes in addition to linear atelectasis in the right lower lobe (coronal view).

The patient was started on a combination of oral letrozole (2.5 mg daily) and oral palbociclib (125 mg daily) for 21 days followed by seven days off in a 28-day cycle. Approximately two weeks after initiating this regimen, the patient presented to the hospital with fever and dyspnea. Her workup revealed neutropenia with a white blood cell count of 1.2 x 10^9^ cells/L and absolute neutrophil count of 0.9 x 10^9^ cells/L (other cell lines were normal). X-ray imaging of her chest was essentially negative. After blood and urine cultures were obtained, she was appropriately started on intravenous antibiotics for the treatment of neutropenic fever. Her infectious workup was negative and her overall condition did not improve after five days of intravenous antibiotic therapy with higher oxygen requirements and worsening respiratory status. A CT scan of her chest was ordered which revealed new ground-glass opacities in the bilateral lungs (Figure 3), suggesting infectious etiology. Bronchoscopy was performed and bronchoalveolar lavage displayed atypical squamous cells suggestive of reactive pattern. She was started on prednisone (1 mg/kg, two times per day). Of note, her palbociclib was on hold since her re-admission. Her respiratory status improved significantly, and she was discharged home in stable condition without oxygen therapy.

## Discussion

Our patient experienced pneumonitis induced by palbociclib. To our knowledge, seven cases (mean age of 65.4 years; age range of 51-89 years; seven females) of palbociclib-induced pneumonitis have been reported in the literature since its approval for metastatic HR+/ERBB2− breast cancer [7-13]. Many of these cases involved patients on combination therapy with fulvestrant, letrozole, and/or zoledronic acid. Presenting symptoms commonly include worsening dyspnea which can occur as early as three months post therapy initiation. Findings include bilateral ground-glass opacities in addition to hypoxia, neutropenia, thrombocytopenia, pulmonary nodular consolidation, interlobular septal thickening, diffuse alveolar infiltrates, and/or pleural effusion. Infectious etiology should be ruled out to diagnose palbociclib-induced pneumonitis. Successful treatment of the condition requires discontinuation of the drug and may involve corticosteroids or home oxygen therapy for several months. It is important to recognize pneumonitis as a potential lethal side effect of palbociclib when administered in order to successfully identify its cause and quickly treat the condition if it arises.

## Conclusions

CDK4/6 inhibitors improve survival of patients with metastatic HR+/ERBB2− breast cancer and thus should be carefully considered, as the treatment of this disease is primarily palliative and not curative. Although rare, pneumonitis can be a side effect of palbociclib. By initiating a prompt workup and plan, we were able to successfully identify and treat this condition in our patient.
